# Modifiable risk factors for post-operative delirium in older adults undergoing major non-cardiac elective surgery: a multi-centre, trainee delivered observational cohort feasibility study and trainee survey

**DOI:** 10.1186/s12877-023-04122-7

**Published:** 2023-07-15

**Authors:** Iain J. McCullagh, Barbara Salas, Andrew Teodorczuk, Mark Callaghan, on behalf of the PODIUM study investigators from INCARN.net

**Affiliations:** 1 Newcastle Hospitals NHS Foundation Trust, Newcastle upon Tyne, UK; 2 Newcastle University, Newcastle upon Tyne, UK; 3 Faculty of Medicine, University of Queensland, Brisbane, Australia; 4 Metro North Mental Health, The Prince Charles Hospital, Brisbane, QLD Australia; 5 School of Medicine and Dentistry, University of Queensland, Brisbane, Australia

**Keywords:** Older adults, Anaesthesia, Age factors, Feasibility, Post-operative, Delirium, Non-cardiac surgery, acute encephalopathy

## Abstract

**Background:**

Post-operative delirium (POD) is an acute brain failure which may occur following major surgery, with serious implications for participants and caregivers. Evidence regarding optimal anaesthetic management for older participants at higher risk of POD is conflicting. We conducted a feasibility study of our protocol in 5 centres to guide sample size estimation and inform future recruitment strategies for a larger cohort study.

**Methods:**

Participants aged over 65 and scheduled for major surgery were recruited. They were assessed pre-operatively for delirium, cognitive impairment, depression, comorbidity, activity levels and alcohol use. Details of management during surgery, all medications and complications were recorded by a trainee-led research team. Participants were assessed for delirium in the immediate recovery period and then on post-operative days 1–4 using the 4 question attention test (4AT) with complications assessed at day 4 using the post-operative morbidity survey (POMS). Primary outcomes were the incident rates of POD. Secondary outcomes were number of eligible patients, recruitment rates and retention rates throughout the study, time required for data collection, preoperative risk factors assessment and daily postoperative delirium assessments. Also to assess the added value of employing the regional trainee research network (INCARNNET) to deliver the study. Specifically, what proportion of patient consent, data collection and post-operative testing is performed by anaesthesia trainees from this group, especially the success of weekend delirium assessment by trainees? A survey was completed at the end of the study by the trainees involved regarding their involvement in the study.

**Results:**

Ninety-five participants were recruited, of whom 93 completed the study. Overall, POD occurred in 9 patients. Of these, three were detected in recovery and six on post-op days 1–4. Median length of stay was 6 days. Recruitment rates were high in all but one site. 59 (62%) participants were consented by trainees and 189 (63%) of post op delirium assessments were performed by trainees. A total of six patients declined the study (in a follow up survey of trainees). Pre-existing cognitive impairment, depression and problem drinking were detected in 4(4.3%), 3(3.2%) and 5(5.37%) participants, respectively. Co-morbidity was common with 55(59%) in class three or four of the geriatric index of morbidity. Overall, from a total of 641 data points, levels of missing data were as follows, site A = 9.3%, B = 13.5%, C = 15.4%, D = 10.9%, E = 11.1% (data could not be completed retrospectively).

**Conclusions:**

A multi-centre observational cohort study of delirium carried out by UK trainee anaesthetists is feasible. Patients are content to undergo day of surgery consent and multiple short questionnaires pre-operatively. Proposed data, especially pharmacological, should be carefully considered for their relevance to modifiable mechanisms that can lead to POD. Future research to enable prognostic modelling of POD should involve large scale cohort studies of enriched populations to capture a higher POD incidence. POD remains a common complication in older persons undergoing major surgery in the UK and studies of interventions are urgently needed.

**Trial registration:**

All methods were carried out in accordance with relevant guidelines and regulations. The study was retrospectively registered with ISRCTN94663125 on 07/02/2018.

**Supplementary Information:**

The online version contains supplementary material available at 10.1186/s12877-023-04122-7.

## Introduction

Post-operative delirium (POD) remains a frequent cause of post-operative morbidity in a variety of types of surgery [1–5]. It is distressing for patients and their caregivers and is associated with significant adverse outcomes including prolonged hospital stay, increased mortality, accelerated cognitive decline and increased rates of institutionalization [6–8]. Major surgery involving older adults is expected to become more frequent with one recent UK projection suggesting that approximately 20% of those over 75 will undergo major surgery per year by the year 2030 [9]. Post-operative delirium and its associated impairment of cognitive function, will inevitably increase as the UK population ages. Effective interventions and preventative strategies are urgently needed, however research in this field remains challenging due to the lack of capacity of potential participants, the number of factors involved and the complexity of potential interventions.

A recent Cochrane review of interventions to prevent delirium in hospitalized patients demonstrated a potential role for multicomponent interventions. In particular there was moderate evidence for depth of anaesthesia monitoring in the surgical subgroup [10]. This was supported by a recent publication on POD which showed that evidence remained variable and proposed that a large cohort study is required in order to clarify the different facets of post-op cognitive abnormalities [11]. Treatment of POD is difficult and varies by suspected aetiology with little evidence for successful drug treatment in particular. Evidence for drug prophylaxis of high risk patients is conflicting with alpha adrenergic drugs such as dexmedetomidine being the most popular candidate agents [12].

In contrast a large body of evidence exists describing predisposing factors. These are known to include older age, alcohol excess, multiple comorbidities, and pre-existing cognitive impairment [13]. In contrast to these non-modifiable or difficult to modify risk factors, we hypothesized that there are modifiable risk factors within current peri-operative practice which could be targets for interventions to prevent POD. Evidence of harm from general anaesthesia is variable. Limited evidence suggests a possible increased risk associated with the use of benzodiazepines and less reliable associations with opioid drugs [14]. There is currently no firm evidence to support one anaesthesia technique over another in those at risk of POD, even in those with high levels of vulnerability such as the hip fracture population [15]. Evidence of benefit from depth of anaesthesia monitoring is also conflicting [16].

In summary, a knowledge gap exists regarding potentially modifiable risk factors for postoperative delirium. This limits our ability to develop effective preventative strategies in target populations. Our intention for the overall project was to identify the specific pharmacological and perioperative factors that modify the risk of postoperative delirium. These factors will then be tested in a treatment trial. The current study is a feasibility study as a starting point for this research. The study was developed and delivered in cooperation with INCARNNET (Intensive Care and Anaesthesia Research Network Northeast Trainees).

## Methods

We conducted a multi-centre prospective cohort study at five general and tertiary hospitals in the Northeast of England. We included participants aged 65 years or older scheduled for major elective non-cardiac surgery, as defined by the Surgical Outcome Risk Tool (SORT) criteria [17]. All participants gave informed consent. Participants with cognitive impairment were included if a personal consultee was available. Urgent or emergency surgery and procedures categorized as cardiac surgery or neurosurgery were excluded, as were participants unable to provide written consent due to language difficulties. Study participants could either be identified by the local study teams in advance e.g. pre-assessment clinic or approached on admission to hospital for their operation, or (most commonly) approached on the day of surgery.

### Outcomes and sample size

Primary outcomes for our study were registered as: incidence of delirium in adults over 65 years of age undergoing major non-cardiac surgery. Secondary outcomes aimed to examine the feasibility and benefits of engaging trainee anaesthetists in performing consent and study procedures, recruitment rates and retention during the study and time taken for study procedures.

The proposed sample size of 96 participants was derived from the recommendation of a sample size of 60–100 for feasibility studies in which an event rate estimate is required for planning purposes. The need to include some subjects who actually had delirium meant that we also took into account the predicted incidence of delirium in this population of 10% (95% CI: 4–16%) [18]. Data are presented as basic statistics.

### Assessments and data collection

All participants completed a series of pre-operative validated questionnaires to assess rates of predisposing factors for delirium. The following tests were performed: Short Portable Mental Status Questionnaire [19], a standardized verbal test of cognition, the Specific Activity Scale (SAS), a measure of functional status [20] and the Geriatric Index of Co-morbidity (GIC), to assess severity and impact of comorbid illness [21]. Participants were also screened for alcohol problems using the CAGE questionnaire [22] and for depression using the Geriatric Depression Scale [23]. The GIC was chosen for its validated accuracy and ease of use, and the SAS for its ease of use by untrained staff. The four-question attention test for delirium (4AT) [24] was performed pre-operatively as a baseline. The 4AT is a widely used and extensively validated delirium screening tool, it is easy to use and has been shown to have high specificity and sensitivity in multiple studies [25, 26]. The 4AT requires no specific training to be used by medical staff who were planned to perform the assessments in he study. All of the other tests relate to clinical assessments which are in areas familiar to trainee anesthetists such as frailty and comorbidity so we did not feel further training was needed (and we felt it would also not be deliverable). Finally, all routine blood tests ,medications, dosages and pre-operative tests such as pulmonary function, echocardiography and cardiopulmonary exercise tests were documented.

The type and timing of the operation performed was noted. All major details of anaesthesia were recorded including all general, central neuraxial or regional anaesthesia performed, and all medications administered documented along with total dosages. For volatile anaesthesia, the drug and mean alveolar concentration (MAC) at the half-way point of the procedure was recorded. In cases using total intravenous anaesthesia (TIVA), target concentrations of propofol and remifentanil were documented. Blood loss and blood transfusion data were collected. All intra-operative data was entered in real time by anaesthesia or research staff from paper records and monitoring equipment.

In the immediate postoperative period (day 0), occurrence of emergence delirium was assessed in the recovery with the 4AT and use of any drug treatment for agitation, pain or nausea was recorded. All drugs administered peri-operatively via any route were recorded including analgesia.

On each of the first four postoperative days (days 1–4) a 4AT was performed if the patient was still in hospital. If any patient was found to have a 4AT score of four or greater, a diagnosis of delirium was made and the treating team were informed. A Post-operative morbidity survey (Clavien-Dindo) [27] was performed on day 4 by the research team if the participant was still in hospital. If patients had been discharged from hospital preventing a 4AT from being completed, we felt it was reasonable to assume that they were not suffering from delirium on that day.

The grade and identity of the individual collecting pre and post op data was recorded. Time taken for each phase of data collection was self-reported. Where possible, pre-operative and post-operative delirium assessments were conducted by the same individual.

All data were entered on to case report forms that were de-identified at sites and entered onto a central database (excel, 2010 version). Analysis was performed using SPSS. (IBM Corp.2017. IBM SPSS Statistics for Windows, Version 25.0. Armonk, NY).

### Survey of medical team

We surveyed medical staff who had taken part in the study using www.smartsurvey.co.uk. The following questions were asked;

1. Which hospital they were involved with during the study,
2. Did they complete good clinical practice training for the first time to take part in this study?
3. Was this the first time they had been involved in performing informed consent for research?
4. did they consent patients in PODIUM.
5. how many if any, refusals to take part were reported.
6. did any patients withdraw from the study.
7. Was being involved in the study enjoyable?
8. Was there adequate time pre-op for consent and questionnaires,
9. How easy to use were the various tools used in the study.
10. How would the rate their overall enjoyment with the study.

## Results

### Clinical outcomes/assessment

Ninety-five participants were recruited between October 2017 and March 2018. Of these, 93 participants completed the study, one participant did not undergo surgery, and one withdrew from the study. 4 sites (all tertiary centres) recruited our planned target of 19 and one site recruited only 3, this meant that one of the other sites was required to restart after a delay to complete the study. Characteristics of the cohort are presented in Table 1.. The mean age of the cohort was 73.4 (67.0-79.8), 61 were male and 32 were female.

**Table 1:** Demographic information about patients, stratified by postoperative delirium diagnosis (POD)

	**No POD (*N*=84)**	**POD (*N*=9)**	**Total (*N*=93)**	***p*** **value**
**Age**				0.777
Missing	4	0	4	
Mean (SD)	73.3 (6.3)	73.9 (7.2)	73.3 (6.4)	
**Gender**				0.505
Male	56	5	61	
Female	28	4	32	
**Anaesthetic**				0.883
Regional alone	26	3	29	
General	58	6	64	
**Surgery**				0.436
Missing	9	0	9	
Open	49	5	54	
Robot-assisted	7	0	7	
Laparoscopic-assisted	17	4	21	
Other	2	0	2	
**ASA Score**				0.954
Missing	9	1	10	
1	1	0	1	
2	41	4	45	
3	32	4	36	
4	1	0	1	
**Specific Activity Scale**				0.543
Class I	47	3	50	
Class II	18	3	21	
Class III	17	3	20	
Class IV	2	0	2	
**Geriatric Index of Morbidity**				0.27
Missing	4	0	4	
Class I	8	0	8	
Class II	21	4	25	
Class III	39	2	41	
Class IV	11	3	14	
No comorbidities	1	0	1	
**CAGE**				0.452
Not clinically significant	79	9	88	
Clinical significant	5	0	5	
**Geriatric Depression Scale**				0.159
No depression	82	8	90	
Depression	2	1	3	
**Portable MSQ**				*
Intact intellectual functioning	80	8	88	
Mild intellectual impairment	3	1	4	
Moderate intellectual impairment	0	0	0	
Severe intellectual impairment	1	0	1	

*No *p* value calculated as no patients in either group with moderate impairment

Our study identified 3 cases of emergence delirium and 6 cases of POD from 93 cases that underwent elective surgery. It is recognized that these conditions potentially have differing aetiologies but for the purposes of this feasibility study they are considered together in Table 2. One further case that was identified to have delirium on POMS questionnaire (despite negative 4AT testing) is thought to be an error and is not included). Characteristics of all participants including those screened positive for delirium are shown in Table 2. Of the participants who screened positive for delirium, six participants received general anaesthesia and three received spinal anaesthesia. Four were scheduled to receive level 2 care post-operatively. Four cases underwent laparoscopic surgery and 5 open surgery. Four participants were ASA 3 and four were ASA 2 with ASA score not recorded in one case.

**Table 2:**
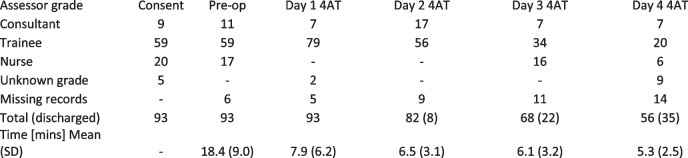
Feasibility data and activity of trainee study team across the study period and overall missing data

**Fig. 1:**
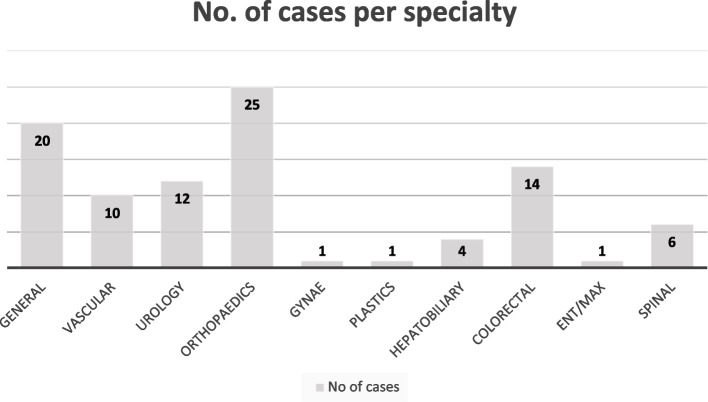
Proportions of surgical specialties in the study

Regarding the whole cohort pre-operatively, cognitive impairment was detected in 4 cases, alcohol problems in 5 cases and depression in 3 cases. Relatively high levels of comorbidity and activity limitation were found with 55 participants in class 3 or 4 of the geriatric index of morbidity and 21 participants in class 2 and 21 in class 3 of the specific activity scale. This is typical of the locale studied which is in an area with high levels of social deprivation. Surgical specialties of the included patients are shown in Fig. 1.

Of the 56 patients remaining in hospital on day four, POMS data revealed that only two were on oxygen, one was receiving respiratory support, six were receiving IV antibiotics, three had been febrile, three were not established on enteral diet, nine had abdominal distension, none were oliguric, one had an elevated serum creatinine, 12 still had urinary catheters in place, three had hypotension. No other cardiac or neurological complications were recorded. Three patients still had delirium on day four, one patient had received a red cell transfusion but no other products were given and there were no wound infections, six patients were receiving parenteral nutrition and 19 had not got back to pre-op mobility levels. Due to the feasibility nature of the study no analyses were performed to assess relationships of these data to delirium.

#### Feasibility outcomes

Feasibility outcome data is presented in Table 2. Informed consent was usually taken on day of surgery, consent was performed by trainee anaesthetists on 59 occasions, by consultants on 9 occasions and by nurses on 20 occasions (missing = 5). Trainee anaesthetists performed pre-op assessments on 59 occasions, consultants on 11 and nurses on 17 (missing = 6). Postoperative 4AT assessments were performed by trainees as intended but with a decreasing proportion over the four days of testing, 79 (84%) on day 1, 56(62%) on day 2, 34 (50%) on day 3 and 20 (36%) on day 4. 16(23%) assessments were performed by nursing staff on day 3 and 6(11%) on day 4. Of the 93 participants, 5 4AT assessments were missed on day 1, 9 on day 2, 11 on day 3 and 14 on day 4. If a patient was discharged the day before a 4AT was due it was deemed unobtainable, but if they were discharged the same day, it was deemed missing, hence some of those deemed missing will in fact be related to discharges.

Our recruitment plan allocated one fifth of recruits (19 participants) to each centre to ensure that trainees in each area had equal opportunities to contribute to the study. Speed of recruitment was variable at the 5 hospitals. Briefly, one site recruited very rapidly and three recruited over 2–3 weeks. One site largely failed to recruit. High recruiting sites recruited the remaining participants after a pause. The most successful site recruited participants rapidly with a large group of trainees and an active local lead. Of the 5 hospital sites that took part in the study 4 recruited at least their target of 19 patients successfully. Overall, from a total of 641 data points, levels of missing data were as follows, site A = 9.3%, B = 13.5%, C = 15.4%, D = 10.9%, E = 11.1%.

All surgery timing data was self-reported. Mean time taken for pre- operative data collection was 18.4 min, for intra-operative data collection 7.5 min and for post –op delirium assessments between 5.3 and 7.9 min. Recording of postoperative drug administration was recorded with a significant degree of variability and represents a large proportion of missing data, but clearly a significant degree of polypharmacy in older participants was present. These data will inform the design of future studies by giving an indication of the proportion of patients on medications of concern. We present the data in summarized form in to illustrate the extent of this polypharmacy with medications potentially related to POD in the cohort in Table 3.

**Table 3:**
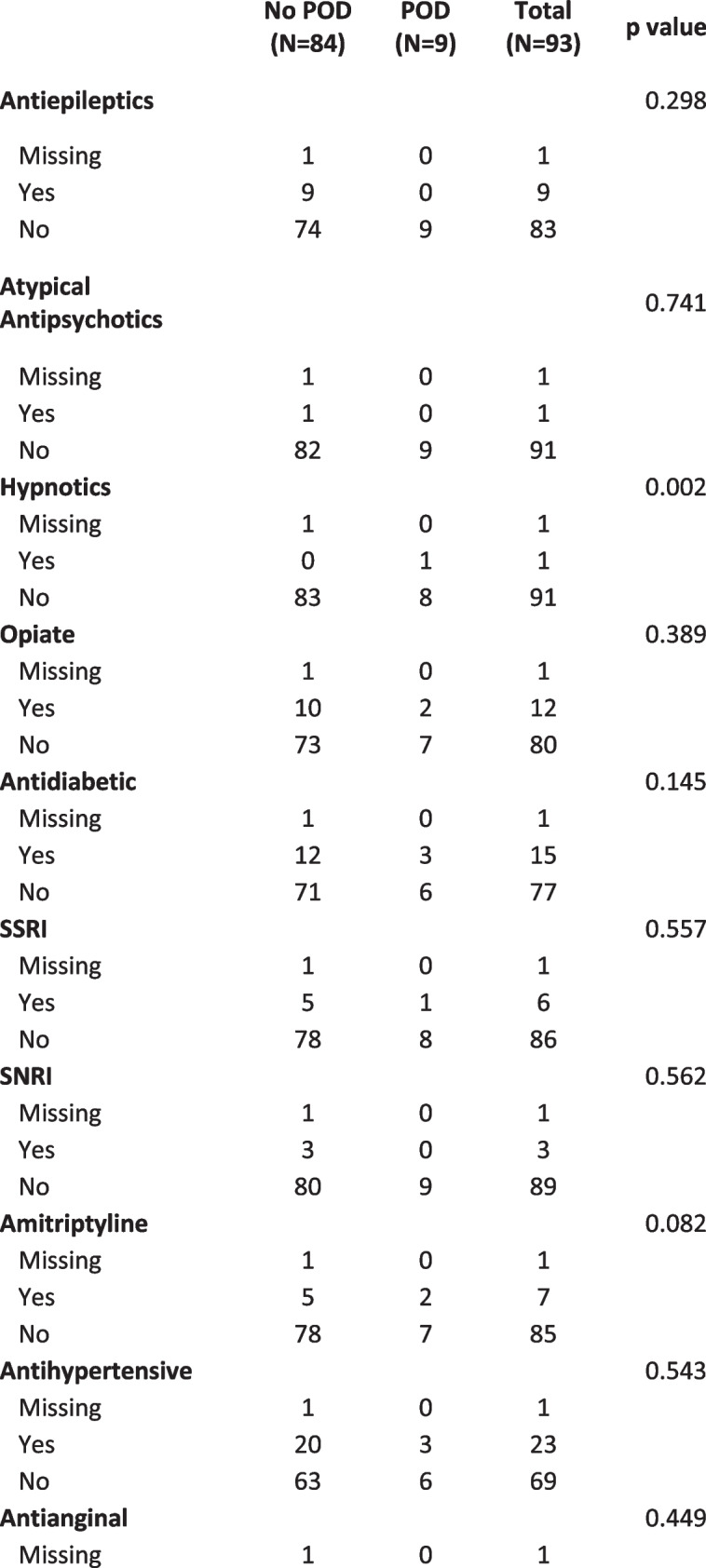
Potentially causative preoperative medication use, stratified by postoperative delirium (POD) diagnosis

#### Survey

The response rate of the survey was 51.6% with 31 trainees involved overall and 16 returns. 15 of 16 trainees reported their experience as positive with one neutral. 6 of 16 trainees gained training in GCP for the first time to participate in the study. 10 of 11 trainees who performed informed consent did it for the first time during their involvement in the study. 11 of 16 felt that there was adequate time to complete consent and pre-op questionnaires on day of surgery. Trainees also rated the difficulty of the various questionnaires; all were rated easy or very easy by between 13 and 16 respondents with the geriatric index of morbidity and specific activity scale the least easy with 2 and 3 trainees respectively saying that these were difficult.

## Discussion

We have shown that UK anaesthesia trainees can carry out repeated peri-operative assessments to enable delirium research. Further, trainees were also able to perform day of surgery informed consent and to establish rates of predisposing patient factors using standardised validated questionnaires.

Our study adds to the emerging literature from a variety of specialties [28, 29] that trainees can, with appropriate support, perform observational research even when this requires repeated patient assessments for complex conditions such as delirium. This is especially important as delirium research is challenging to undertake. Pre-operative assessments were performed reliably and comprehensively. We deliberately avoided longer term follow up as we did not feel that trainees would be able to manage this without significant supervision and infrastructure. We found the most effective method to be rapid large-scale recruitment over a short time-period with research nurse support. We would use this approach in any large scale follow up study. We are confident that the majority of missing data was either due to patients already having been discharged or because the suggested dataset was too large to be gathered in such a short time. We believe an eCRF would also reduce problems with missing data as such forms can use digital means to ensure completion before submission.

Our study found a rate of incident delirium and emergent delirium of 6.5% in an elective non-cardiac, non-neurosurgical population aged over 65. This is at the lower end of previously published estimates of around 31% in the hip fracture population [30] and between 23 and 25% in the recent ENGAGES trial [31]. The reasons for this low incidence likely relate to the high numbers of participants in our study with short lengths of stay in hospital. This suggests that despite all surgery being categorised as “major” according to the SORT tool, the likelihood of delirium at the outset in this population was relatively low. Also, we recruited participants aged over 65 rather than aged over 70 as used in some other studies. That said, the detected rate of POD in this cohort would still result in more than 30,000 older adults suffering post op delirium per year in the UK and suffering negative outcomes as a result.

### Weaknesses

We assessed for delirium on a single point each day and we did not include a daily chart review in our study protocol, these issues may have resulted in missed cases of delirium. Delirium assessments were limited to the first four postoperative days; it is possible that in those with longer stays, we could have missed cases of delirium but less likely that these cases were related to the index operation. Whilst it is possible that additional cases of delirium could also have occurred in those discharged on the same day as assessment was missed, we believe it is unlikely. We recognize however that mild cases are not always obvious without specific assessment. In the UK patients with newly diagnosed delirium post-operatively would not normally be discharged from hospital unless a clear cause was identified and clear improvement was observed but this may not be the case in other jurisdictions. Missing data is a potential issue but most routine data could easily have been collected by research teams post hoc had we requested this, we did not do this in order to maximize our understanding of the research process.

Trainees were slightly less successful in collecting complete post-operative medication information than in other aspects but this was felt to be due to the nature of the case report form and the extensive level of detail demanded which included dosage, frequency and all associated medications. In retrospect this detail was out of proportion to the time resources available to the trainees involved.

### Future research

UK anaesthesia trainees have shown recently with the DALES study (drug allergy labeling in elective surgery) [32] that using “bring your own device” technology with easy to complete digital case report forms, can be effective in simple large-scale observational studies with small data sets. This has relevance for recent SNAP 3 study (Sprint National Audit Project 3) [33] which is similar in design and is assessing frailty and delirium. Hopefully, new developments in formalizing trainee involvement in research such as the NIHR associate PI scheme will ensure that SNAP 3 is successful, as it will mirror what we found to be the most effective strategy of recruiting participants rapidly to focus follow up efforts over a short period of time. We are confident that trainee anaesthetists could collect our required data with small modifications to our approach to reduce missing data.

At the forefront of any future work should be the clear understanding that POD is a clinical syndrome that is difficult to assess and diagnose, with heterogeneity of presentation, cause, and duration for which there is currently no effective treatment. We have formed a UK wide research group that has recently published a series of systematic reviews to enable us to establish the full range of interventions and predisposing and precipitating factors that have previously been studied in relation to POD [34, 35]. We will carefully consider the learning from our feasibility study and from SNAP 3 which will enable us to focus intervention studies on patients at higher risk of POD [36]. Adequate funding and methods for increasing certainty of delirium diagnosis will be in place for any follow up study. This study will be performed as part of a comprehensive program of study of POD in the UK. We will develop carefully selected interventions in line with UK guidelines [37] and according to the framework put forward by the MRC [38] and test these interventions in an enriched cohort of older adults undergoing major surgery in a subsequent trial.

## Conclusions

We identified an incidence of POD of 6.5% in adults 65 and older undergoing major surgery. Trainee anaesthetists carried out study tasks effectively. A large-scale trainee delivered cohort study of post-operative delirium is feasible, but the longitudinal nature of the study means that a short time-period should be used to maximise impact and loss of momentum and ensure high quality outputs. Delirium incidence in our cohort was low so future studies should target groups at higher risk. This study could use direct, electronic means of data collection and should include routinely collected data where possible. Fidelity of delirium assessment should be strengthened by using expert confirmation and notes review.

## Supplementary Information

**Additional file 1.**

## Data Availability

The datasets generated and/or analysed during the current study are not publicly available due to permission for this not being sought from participants or the ethics committee but are available from the corresponding author on reasonable request.
