# Supplementary material for: Modifiable risk factors for post-operative delirium in older adults undergoing major non-cardiac elective surgery: a multi-centre, trainee delivered observational cohort feasibility study and trainee survey
Source: BMC Geriatr. 2023 Jul 15;23:436. doi: 10.1186/s12877-023-04122-7 (PMC10349417; doi:10.1186/s12877-023-04122-7)
Supplement: Supplementary file 1 — Additional file 1. [file 12877_2023_4122_MOESM1_ESM.docx]

**Modifiable Risk Factors for Post-operative Delirium in Older Adults undergoing Major Non-cardiac Elective Surgery: A Multi-centre, trainee delivered Observational Cohort feasibility study and trainee Survey.**

Appendix 1

Contributing team involved in conduct and delivery of the study.

Patrick Chiam^1^, Kerry Colling^1^, Jennifer Noyes^1^, Rachel Horner^2^, Joanne Knight^3^, Will Udall^8^, Abigail Butler^8^, Allan Pang^4^, Thomas Livingstone^5^, Michelle Shaw^1^, Mark Callaghan^8^, Michael James^8^, Ben Goodman^8^, Claire Marie Agius^8^, Abigail Harrison^8^, Leigh Dunn^8^, Sneha Prasad^8^, Christine Wood, Amy Fox^8^, Annie Bowe^3^, Barbara Prysbysz^8^, Leonie English^5^, Lucy Venyo^6^, Peter Lawley^5^, Ross Dryburgh^8^, Sarah Welch^8^, Sophie Cronin^2^, Steven Traill^8^, Stephen Robb^2^, Sophie Curtis^2^, Adrian Taylor^5^, Robert Darke^8^, Stephen Mowat^5^, Patrick Hayes^7^, David Buckley^8^, Sarah Higgin^8^, Edel McCauley^1^

1 South Tees Hospitals NHS Foundation trust 2 Sunderland and South South Tyneside Foundation trust 3 Gateshead Health NHS Foundation trust 4 Leeds ADACC 5 Northumbria Healthcare NHS Foundation trust 6 North Tees and Hartlepool Hospitals NHS Foundation trust 7 University Hospitals Sussex NHS Foundation trust. 8 Newcastle Hospitals NHS Foundation Trust.
